# Joint positioning sense, perceived force level and two-point discrimination tests of young and active elderly adults

**DOI:** 10.1590/bjpt-rbf.2014.0099

**Published:** 2015-08-07

**Authors:** Priscila G. Franco, Karini B. Santos, André L. F. Rodacki

**Affiliations:** 1 Departamento de Educação Física, Universidade Federal do Paraná (UFPR), Curitiba, PR, Brazil

**Keywords:** physical activity, aging, proprioception, movement

## Abstract

**Background:**

Changes in the proprioceptive system are associated with aging. Proprioception is important to maintaining and/or recovering balance and to reducing the risk of falls.

**Objective:**

To compare the performance of young and active elderly adults in three proprioceptive tests.

**Method:**

Twenty-one active elderly participants (66.9±5.5 years) and 21 healthy young participants (24.6±3.9 years) were evaluated in the following tests: perception of position of the ankle and hip joints, perceived force level of the ankle joint, and two-point discrimination of the sole of the foot.

**Results:**

No differences (p>0.05) were found between groups for the joint position and perceived force level. On the other hand, the elderly participants showed lower sensitivity in the two-point discrimination (higher threshold) when compared to the young participants (p < 0.01).

**Conclusion:**

Except for the cutaneous plantar sensitivity, the active elderly participants had maintained proprioception. Their physical activity status may explain similarities between groups for the joint position sense and perceived force level, however it may not be sufficient to prevent sensory degeneration with aging.

## Introduction

Physiological changes associated with aging lead to decreased functionality and reduced independence^1^. Physiological changes include progressive reduction of the visual, vestibular, and proprioceptive systems that are essential to maintaining and/or recovering balance and reducing the risk of falls^2^. Falls are a serious health problem^3^, as its complications are the leading cause of hospitalization and death among individuals over 65 years^4^. Moreover, falls directly affect the quality of life of elderly adults because of their influence on lifestyle and health^5^.

Changes in the central and peripheral nervous systems decrease the ability of the elderly to identify several stimuli in the environment and to select appropriate responses^6^. Losses in the proprioceptive system reduce the ability to continuously monitor motor sequences and interfere in coordination and balance. Proper functioning of the proprioceptive system is crucial to adequate responses and correct actions of the segments involved in the movement^7^. Thus, these changes can influence the level of physical activity and functional capacity needed to perform daily tasks independently^2^. Furthermore, proprioceptive deficits may retard the perception of perturbations that require quick responses, e.g. stumbling and falling, thus leading to longer response times and decreasing the ability to restore the balance.

On the other hand, regular physical activity has been said to contribute to improve proprioception and therefore reduce falls with aging^8^. Thus, this study aimed to compare the performance of young and active elderly participants in the following proprioceptive tests: (a) perception of joint position; (b) perceived level of strength; and (c) two-point discrimination on the sole of the foot. It was hypothesized that the elderly will present worse performances than the young group.

## Method

Twenty-one young adults and 21 active elderly adults volunteered to participate in the study. For both groups, inclusion criteria required classification as physically active and exclusion criteria included: absolute or relative contraindications to the protocols applied in the study, history of recent joint surgery, use of prostheses, orthoses or assistive devices, chronic heart, lung or skin problems, no physical disability, sufficient mental ability to understand the test protocols, and ability to perform daily activities independently. These variables were obtained based on self-report assessment. Participants received information about the procedures and signed an informed consent form. The experimental procedures in this study had the approval of the Research Ethics Committee of *Universidade Federal do Paraná* (UFPR), Curitiba, PR, Brazil (approval number CEP/SD 986.111.10.08; CAAE 0063.0.091.000-10).

### Instruments and procedures

Participants were required to answer the International Physical Activity Questionnaire (IPAQ) that revealed an active physical status. Participants also performed the following tests: two-point discrimination, joint positioning sense, and perceived force exertion. The tests were applied in this order in an attempt to minimize possible fatigue effects.

### Joint positioning sense test

**Figure 1.**
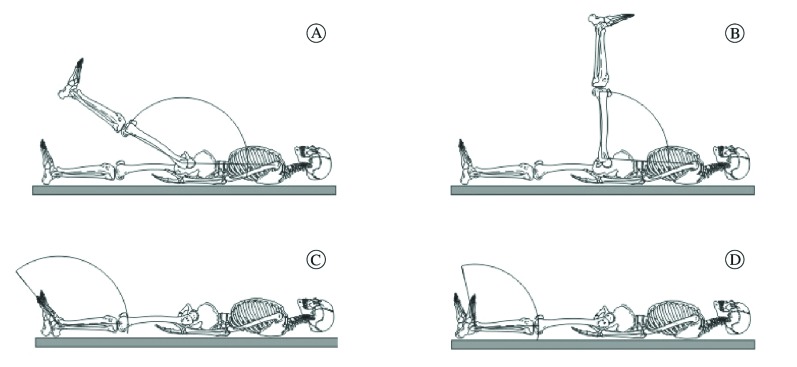
Schematic representation of the hip and ankle joints during the joint positioning test. Narrow and wide range flexion positions during the joint positioning sense test of the hip (top panel A and B) and ankle (lower panel C and D).

The joint positioning sense assessment was performed with the participants positioned in a supine posture and had determined their maximum passive flexion range of motion of the right hip and ankle. The hip and ankle joints were selected because they play a relevant role during the maintenance and recovery of balance^9^. The range of motion was determined in the anterior aspect taking into account the angle between the thigh and the trunk for the hip angle and the foot and the shank for the ankle. In both joints, a fully extended position corresponded to 180º. A goniometer (positioned at the joint center) helped to determine the maximal ranges of movement. Then, the relevant joint was moved during 5s through a "narrow range" (i.e., less than half of the maximum range) or through a "wide range" (i.e., more than half the maximum range) in a random order. These "narrow" and "wide ranges" were chosen as postures that should be replicated while repositioning the segments, i.e., as a target posture. The narrow and wide ranges of the hip were 136.95º±8.61º and 110.10º±7.62º for the elderly and 138.76º±8.68º and 95.67º±10.65º for the young group, respectively. The narrow and wide ranges of the ankle were 143.00º±8.41º and 119.95º±7.28º for the elderly and 140.86º±7.36º and 110.43±6.91º for the young group, respectively. Immediately after determining the target angles in each condition ("narrow" and "wide" ranges), participants were allowed three attempts to reproduce each position. Participants were deprived of visual information during the test. The target position in each trial was photographed to determine the variations in joint positioning. Figure 1 shows a schematic representation of the postures.

The photographs were taken on the right side of the body using a camera (Sony Cyber Shot, 4.1), perpendicularly positioned at approximately 1.2 m from the right sagittal plane and with the focus pointing to the center of the relevant joint. A set of markers (9.5 mm diameter) were previously placed on the skin, over the following landmarks: (1) acromion; (2) greater trochanter; (3) lateral condyle of the femur; (4) lateral malleolus; (5) base of the fifth metatarsal. The discrepancy between the average of three trials and the target position was used to determine the ability to reposition the body segments.

### Perceived force level test

**Figure 2.**
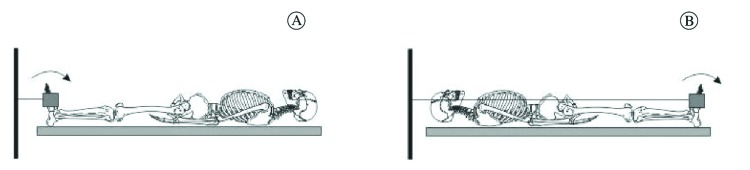
Schematic representation of the ankle during the perceived force level test. Participants performed maximal isometric voluntary ankle dorsiflexion (A) and plantar flexion (B), then contraction equivalent to 10% and 20% of the maximal with visual feedback by numerical computer, and finally the performances reproduced a target force.

The perceived force level test was accomplished by asking the participants to reproduce a percentage of the force determined during a maximal isometric voluntary contraction only around the ankle joint (Figure 2). The maximal isometric voluntary contraction was performed in a supine posture, and participants were required to perform ankle plantar flexion and dorsiflexion at maximum strength. Then, participants were allowed a period of familiarization with the protocol in which they were demanded to reproduce a target force. A numerical computer display provided visual feedback. They were deemed familiar with the test when variations were less than 10% of the target value.

In the perceived force level test, participants were asked to perform an isometric contraction equivalent to 10% and 20% of the maximal isometric voluntary contraction. The ankle was positioned at approximately 90º, and tests were executed in a random order. Visual feedback was provided once in each test condition, and then two trials without verbal or visual information were performed. Participants signaled as soon as they believed they had reached the desired force level. The discrepancy between each target condition (10% and 20% of maximum isometric voluntary contraction) and the mean of three perceived values of the respective condition were used to indicate the ability to generate specific force levels.

The force was determined in the dominant side using a load cell (Kratos, Model IK - 1C, Brazil - 500 Kgf capacity and 0.1 Kg resolution). The load cell was perpendicularly connected to the foot by steel cable attached to a Velcro strap.

### Two-Point discrimination test

The two-point discrimination test was described by Franco et al.^10^ who confirmed the intra-rater reproducibility of the test (range from 0.7 to 1.5%). The test was carried out on the sole of the right foot around the region of the first metatarsal. A two-point discriminator device (Touch - Test TM, model NC12776, measures 1 to 25 mm, North Coast Medical, Inc., Ireland) was applied perpendicularly to the sole and the weight of the device was applied simultaneously to both ends. Participants were asked whether one or two ends were in touch with the skin. The distance between the tips was reduced in increments of 1 to 2 mm, until participants were unable to distinguish whether two points were being applied to the skin. Further details about the two-point discrimination test can be found elsewhere^10^.

### Statistics

Initially, data was processed using standard descriptive statistics (mean and standard deviation). Then, the Shapiro-Wilk test was applied and confirmed data normality. The Levene test confirmed data homogeneity. The data that did not fulfill the criteria for normal distribution were normalized using logarithmic, quadratic or exponential functions. Proprioception was compared between groups using a t-test for independent measures. Bonferroni's approach was applied to adjust significance level due to multiple comparison effects. Statistical tests were performed with Statistica software (StatSoft Inc.^(r)^, version 7.0), and the level of significance was set at p≤0.05.

## Results

The sample was composed of 42 participants, who were allocated to two groups: young group (24.62±3.88 years; 64.43±9.53 Kg; 1.69±0.09 m) and elderly group (66.95±5.55 years; 65.34±13.67 Kg; 1.56±0.08 m). The participants were classified as active by the IPAQ and were able to understand and perform the tests.

The joint positioning test was not able to distinguish young from the elderly (p>0.05). The absolute errors in repositioning the hip and ankle joints and the percentage differences between the groups are shown in Table 1.

**Table 1.** Relative difference between groups and absolute discrepancies between target and intended positions during the joint positioning test performed at narrow and wide hip and ankle ranges of elderly and young subjects.

	Range	Elderly (n=21)	Young (n=21)	Difference (%)	p
Hip	Narrow	7.29±5.80°	5.31±3.45°	37.29	0.19
	Wide	4.21±2.85°	3.15±2.29°	33.65	0.19
Ankle	Narrow	3.56±2.87°	2.32±1.91°	53.45	0.11
	Wide	3.33±2.92°	3.33±2.46°	0.00	0.99

No differences were observed between groups in the level of perceived force test. However, differences were borderline and indicated that there is a trend of a reduced perception of force between groups when the ankle was tested at 20% of maximum voluntary isometric contraction (p=0.06, dorsiflexion and p=0.09, plantar flexion).

Although no differences were found between groups (p>0.05), errors in perceived force increased when higher force levels were required. The perceived force errors and the percentage difference between groups are shown in Table 2.

**Table 2.** Relative difference between groups and absolute discrepancies between target and executed force exertions during the perceived force level test of the plantar flexor and dorsiflexor muscles of elderly and young subjects.

		Elderly (N=21)	Young (N=21)	Difference (%)	p
Plantar flexion	10%	1.87±1.42	1.54±1.08	21.43	0.40
	20%	2.59±1.85	1.87±1.62	38.50**	0.09
Dorsiflexion	10%	1.59±2.24	1.66±1.06	4.22	0.34
	20%	2.82±2.44	1.70±1.10	65.88*	0.06

* p=0.06;

** p=0.09.

The elderly group showed a higher threshold in the two-point discrimination test of the sole of the foot when compared to the young group (p=0.05; 13.43±2.62 mm and 6.19±2.09 mm, respectively).

## Discussion

The main outcomes of the study revealed no differences between active elderly and young subjects in the joint positioning sense and perceived force level tests. In contrast, the two-point discrimination test showed that young subjects are able to better identify stimulus applied to the sole of the foot.

The joint positioning sense test evaluated the ability of subjects to perceive their body arrangement in order to reproduce a target posture. Passive segment repositioning tests are less complex and may not represent the real conditions performed in daily life actions, where movements should be intentionally controlled to meet the demands and interact with the environment accordingly. Daily actions are controlled based on proprioceptive feedback^11^ during movement rather than when the segment is positioned in a particular posture, as in passive tests. Therefore, the active joint positioning sense tests seem to be more suitable and specific for functional assessments than passive tests.

When trying to reproduce a previously established position, the elderly participants showed a slight (non-significant) tendency to present larger errors than did their young counterparts. Several arguments may explain the similarities between the elderly and young groups. Physical activity has been reported to modulate the functional state of the muscle and therefore influence proprioception^8^. Franco and Rodacki^12^ found that differences in proprioception between young and elderly subjects were present only when active young subjects were compared to sedentary elderly subjects. Tsang and Hui-Chan^13^ also identified the influence of physical conditioning on the ability to identify segment postures around the knee joint while comparing active and sedentary elderly subjects. It may be argued that physical activity demands continuous muscle involvement, which is able to slow down the effects of advancing age on muscle tissue^14^. Thus, the results of this study indicate that the perception of joint repositioning may be more associated with the level of physical activity than chronological age itself. Several studies have shown better performance after a training program in tasks that are heavily dependent on proprioceptive information, such as postural control. Improvements in postural control have been reported after a training program designed to stimulate sensory afference when compared to other training programs that involved strength as the primary goal^2^.

The elderly showed greater difficulties in repositioning the lower limbs in narrow range postures during hip flexion and ankle dorsiflexion. The increased rigidity of the tissues surrounding the joints that is typically present in the senescence process may have played a role and reduced the maximal joint range of motion^15^. Therefore, positions that required wide ranges may have imposed a greater distention of the surrounding tissues and facilitated afferent sensory feedback. Thus, it may have produced a greater sensory stimulus that resulted in a small joint repositioning error^12^. This may be true specifically for the ankle joint, where narrow ranges tend to be near the maximum joint range of motion. Petrella et al.^16^ reported significant differences in the ability to actively reproduce a given knee angular position between young, active elderly and sedentary elderly subjects and reinforced the arguments that afferent sensory decline occurs and that physical activity plays a relevant influence on proprioceptive functioning.

Petrella et al.^16^ reported differences when young and active elderly subjects were compared, however performing the joint positioning sense test while standing may have influenced results as balance requirements are known to occur in the elderly^17^. Thus, one may argue that positioning the knee segment in a defined position in a standing posture may have included additional balance demands. The supine posture adopted in the present study is likely to be less susceptible to the influence of balance requirements and represent a more realistic approach of the active joint positioning sense. Therefore, differences in methodological approaches may at least partially explain contrasting results.

The perceived force level test was designed to evaluate the ability to perceive and voluntarily control low force contraction levels of the dorsi- and plantar-flexor muscles. The elderly presented greater variability than the young in all perceived force level test variables, indicating greater difficulty in controlling the production of a given level of force, regardless of the intensity of muscle contraction^18^. These findings are relatively common in the literature using discrete^19^ or continuous isometric contractions^20^. The results of the present study are also consistent with the findings that variability increases during contractions that require low force although most studies have used very small MVIC percentages (ranged from 2 to 10%^21^^,^ 22). The percentages of 10 and 20% MVIC were selected to represent the more functional demands of daily tasks.

The results indicated that force perception errors were reduced in the elderly group when loads of 20% MVIC were applied in comparison with the young group. It may be argued that very small levels of force (e.g., 10% MVIC) are unusual and difficult to control for both groups. The torque observed around the ankle joint is usually greater than 20% of the maximal torque. For instance, Billot et al.^23^ reported that during simple tasks such as one-legged standing, the elderly subjects created a torque around the ankle of approximately 45% of their maximum, while the torque observed in young adults during the same task was 15% of the maximum. Hortobágyi et al.^24^ have also demonstrated that elderly subjects present larger relative knee joint torques when compared to young subjects while rising from a chair. Thus, subjects may be more familiar with torques within their daily range than with very small torques. In addition, there is evidence that training can remove differences at low %MVCs^22^. Therefore, the active status of the subjects may have masked the differences between elderly and young participants.

The elderly group showed lower ability to discriminate two-point pressure in the sole than the young group, although no peripheral sensory disorders were self-reported or identified. The sensitivity responses of the elderly group can be compared to that reported in type II diabetes patients when a similar methodology was applied^25^. These findings are in agreement with others that have reported sensory degeneration in the elderly^26^. Indeed, similar results are also found in other studies that applied the two-point discrimination test to the hand of elderly subjects^27^.

The afferent signals from cutaneous receptors in the sole provide spatial and temporal information and influence balance and stability. Thus, they play a significant role when compensatory actions are required to sustain an erect posture^28^. Indeed, Toledo and Barela^17^ observed that the more degenerated the proprioceptive system of the elderly subjects was, the greater the oscillations of the center of pressure, which reinforced the idea that balance is influenced by the proprioceptive information of the sole of the foot. Others have reported that the ability to discriminate two points in the sole (i.e. preserved sensory response) is associated with a lower incidence of falls^29^.

In the present study, the ability to discriminate two points was compared between the elderly and young groups. The results suggest that physical activity may have had a positive effect on proprioception. Santos et al.^30^ reported increased tactile sensibility in the sole and reduced anterior-posterior oscillation in diabetic women after a 12-week training program designed to improve proprioception. These results were not confirmed in the present study, in which only the physical activity level was considered. It can be argued that maintaining physical activity may not be sufficient to elicit adaptive responses from the sensory system, which may require more specific or intense stimuli.

The results must be viewed with caution, as a group of sedentary elderly subjects was not included. In addition, the sample was relatively small and a larger sample could influence the results.

## Conclusion

The results of the joint positioning sense and perceived force level tests showed similar results between elderly and young subjects. They also suggest that maintaining a physical activity level may not be sufficient to improve the sensory system and elicit responses to stimuli applied to the sole. Thus, the reduced sensibility during the two-point discrimination test of the soles explains the poorer performance by the elderly group in comparison with the young group, and it was interpreted as the result of the degenerative senescence process. Thus, it can be inferred that physical activity may influence the effects of ageing of joints and muscle proprioceptors, but not the sensory system of the soles of the feet. Intervention studies including physical activity programs are required to determine the effects of proprioception.
